# ‘The Drugs Did For Me What I Couldn’t Do For Myself’: A Qualitative Exploration of the Relationship Between Mental Health and Amphetamine-Type Stimulant (ATS) Use

**DOI:** 10.1177/11782218211060852

**Published:** 2021-12-06

**Authors:** Liam Patrick Spencer, Michelle Addison, Hayley Alderson, William McGovern, Ruth McGovern, Eileen Kaner, Amy O’Donnell

**Affiliations:** 1 Population Health Sciences Institute, Newcastle University, Newcastle upon Tyne, UK; 2 Department of Sociology, Durham University, Durham, UK; 3 Department of Social Work, Education and Community Wellbeing, Northumbria University, Newcastle upon Tyne, UK

**Keywords:** Amphetamine-type stimulants, mental health, substance use, treatment, qualitative research

## Abstract

Substance use and mental ill health constitute a major public health burden, and a key global policy priority is to reduce illicit and other harmful substance use. Amphetamine-type stimulants (ATS) are the second most used class of illicit drugs and a range of mental health issues have been documented amongst users. This paper explores the relationship between mental health and ATS use, through a thematic analysis of qualitative interviews with n *=* 18 current and former ATS users in England. The findings are presented by trajectory point of; (1) Initiation of ATS use; (2) continued and increased ATS use and (3) decreased and remitted ATS use. This work helps to develop understanding around the complex and bi-directional relationship between ATS use and mental health. Many ATS users lead chaotic lives and engage in multiple risk behaviours, however there is a need to better understand and conceptualise the dynamic interaction between different individual, social, environment and cultural factors that determine individuals’ mental health and substance use. There is no ‘one size fits all’ approach to prevention and treatment, and these findings highlight the need for more joined-up, tailored and holistic approaches to intervention development.

## Introduction

Substance use and mental ill health constitute a major public health burden amongst the population^1^ and globally a key policy priority is to reduce illicit and other harmful substance use.^2^ Amphetamine-type stimulants (ATS) such as amphetamine, methamphetamine and methylenedioxy-methamphetamine (MDMA/Ecstasy) are the second most commonly used illicit drug worldwide, with an increase in production and use seen in recent years.^3,4^ In 2018 lifetime prevalence of ATS use was estimated at 13.5 million (4.1%) for MDMA, and 11.9 million (3.6%) for amphetamines amongst 15 to 64 year olds in Europe,^5^ with an estimated 1 in 11 adults in the UK reporting consumption at some point in their lives.^5,6^ In England and Wales, the social and economic cost of illicit drug use, including policing, crime and healthcare is £10.7 billion per year.^7^ However, the societal impacts are further reaching, with the practice of problematic substance use in our communities leading to increased levels of blood-borne viruses, drug-related deaths and drug-driven crime. Mental ill health, domestic abuse, offending and bereavement are often associated with problematic substance use, impacting upon substance users themselves, their children, and other significant individuals.^7^ There is evidence of discrepancies in care quality for those with co-occurring mental health and substance use disorders, highlighting the need for more to be done to reduce this inequality, and support these individuals.^8^

ATS have sometimes been viewed as recreational or ‘safe’ drugs, with users reporting perceived positive effects, such as increased sociability, energy, talkativeness and positive mood, whilst underestimating and less frequently reporting the negative social and health effects.^9,10^ However, ATS use has been identified as a risk factor for poor mental health, and even serious mental illness,^11,12^ with a range of issues documented amongst users, including depression, anxiety and changes in mood.^13^ Heavy or prolonged use of some ATS, such as methamphetamine or novel psychoactive substances (NPS) have been noted to impact negatively upon individuals’ physical health, mental health and neurological functioning,^14^ with some users experiencing transient or persistent psychotic symptoms, agitation and insomnia.^11,15-18^ Short-term issues may be due to the acute effects of ATS intoxication, however prolonged effects may be related to withdrawal.^1^ The relationship between, and co-occurrence of, mental health issues and substance use is complex and bi-directional in nature, and both may have a common set of underlying and often compounded causes located across individual and structural factors.^19-21^ It has also been argued that individuals who experience mental ill health problems are more likely to be or become dependent on substances^22^ and similarly, individuals who misuse substances appear to be more likely to develop or suffer from mental health problems.^23^ Determining whether the mental health issue or problematic substance use occurred first is further complicated because the relationship between psychological symptoms and substance use is temporal, meaning that many individuals may experience both substance-induced and substance-independent mental health issues throughout the course of their substance use careers.^24,25^ Despite existing research having established a link between ATS use and mental health issues, there is little known about the order of onset and the implications of this for treatment.^26,27^

The mixed-methods European ‘ATTUNE’ study aimed to address a gap in knowledge on what shapes ATS use across the life-course; how to prevent and treat harmful ATS use and what influences different trajectories of consumption through individuals’ lives. Prior to this, there was limited understanding around which factors lead to increased ATS use, or what could help to facilitate decreased use, or desistance.^28,29^ This paper reports on a sub-sample of ATTUNE qualitative data collected from interview participants in the North East of England, and aims to specifically explore individual experiences of, and perspectives on the relationship between mental health and ATS consumption. This is important, as there has been little investigation of the relationship between ATS use and co-occurring mental health problems,^30^ the management of which continues to be challenging for clinicians.^31^ There is not yet an established substitute pharmacological treatment for dependent ATS users.^32^ Whilst some considerable psychological and psychosocial intervention developments have been made in recent years,^33,34^ treatment services are predominantly based on a medical model, geared towards alcohol and opioid dependencies, which ignores the social and environmental drivers of substance use, and characterises people who use substances as requiring external control.^35^

## Methods

### Design

Qualitative research is recognised as enabling in-depth analysis of socially situated experiences, and can help to provide insight into otherwise unknown practices; ensuring better-informed public health policy decisions through the identification of optimal opportunities for intervention, prevention and treatment.^36^ The qualitative phase of the ATTUNE study used in-depth semi-structured interviews with ATS users and non-users, to provide an in-depth understanding of the lived experiences of participants and the factors which shape different trajectories of ATS use through the life-course. Ethical approval was granted by Newcastle University, Faculty of Medical Sciences, Ethics Committee (REF: 01204/2016) on 8th September 2016. Interviews were conducted between February and July 2017.

### Recruitment and sample

Interviews were conducted in the North East of England, a region that has experienced substantial economic decline since the 1980s, with high levels of unemployment, disability and economic inactivity.^37,38^ To be eligible to participate, individuals must: have first used, or had the opportunity to use ATS at least 5 years previously; be over 18 years of age; have lived in the North East of England and have appropriate verbal and cognitive skills to provide informed consent. Relevant organisations, such as homeless charities, substance use services and probation teams across the North East region signposted potentially eligible participants to study information. Flyers and posters advertising the study were distributed amongst local community spaces, including cafes, bars and libraries. The study was also advertised online, through social media and via national academic, policy and practice community networks. ATS use was categorised as current (use within the past 12 months) and former (use over 12 months ago). Users were further categorised according to whether they were: current/former dependent users (positive [⩾4] Severity of Dependence Scale^39^ [SDS] score); current/former frequent users (at least 10 days use, but not SDS positive); or current/former non-frequent users (less than 10 days use in a 12 month period).^28,40^

### Data collection

Potential participants were provided with an information leaflet, explaining that their participation was confidential and anonymous, and were given the opportunity to ask any questions. If the individual was willing, able and eligible to participate, then written informed consent was obtained. The interviews were conducted face-to-face by 1 of 3 members of the research team (MA, LS, WM). A semi-structured interview topic guide was used, with questions and follow-up probes related to family, physical and mental health and key turning points in drug use trajectories, namely initiation, continuation, decrease, desistance and relapse of ATS use. The interviews were digitally audio-recorded and transcribed verbatim. We continued data collection until there was maximum diversity across the sample in terms of ATS use, age, gender and socioeconomic status^41^; and data saturation was believed to have been achieved, as identified through repetition of responses and sufficient data to answer the research questions.^42^ Participants received a £10 shopping voucher incentive for participating. Interviews lasted between 18 and 106 minutes (mean = 48 minutes).

### Analysis

During an initial framework analysis^43^ of the whole sample of n = 70 interviews, a sub-sample of n *=* 18 current and former ATS users were identified. The relationship between mental health and ATS consumption was already an identified knowledge gap in this area,^30-32,35^ therefore further analysis was conducted using a thematic approach^44^ by LS, to focus specifically on this issue. It is however important to note that this does not mean that the remainder of the whole sample experienced no mental health challenges, just that the focus here was on those for whom these challenges were a recurrent theme and central issue. Data were coded iteratively, using a combination of inductive and deductive approaches and emergent themes were discussed with AOD and HA. Bronfenbrenner’s ecological theory was drawn upon in order to help better understand and unpack patterns and risk factors associated with co-occurring substance use and mental health.^45^ This theory was used to guide the interpretation of data; and states that individuals are shaped not only by individual factors, but to a great extent by the social, economic and physical environments in which they are situated.^44^ Considering interactions within and between these different ecological systems offers a way to focus on both intrapersonal and environmental factors, and the dynamic interplay between these factors in determining behaviours and health outcomes,^46,47^ which was useful to consider whilst exploring the complex issue of interest. All data were coded and organised using NVivo 11.

## Findings

The 18 current and former ATS users interviewed were aged 20 to 45 (mean age = 33.2 years; 56% male, 44% female). The characteristics of the participants are presented in Table 1. The findings are categorised in relation to 3 key turning points in ATS use careers: (1) initiation; (2) continued and/or increased use and (3) decreased and/or remitted use, exploring the interaction between mental health and ATS use at each turning point. Each category is presented in greater detail below, with verbatim quotations to illustrate the findings.

**Table 1.** Participant characteristics (N = 18).

	N
Gender	
Male	10 (56%)
Female	8 (44%)
Age range (20-45; mean = 33.2; SD = 8.5)	
20-30	7 (39%)
31-40	7 (39%)
40+	4 (22%)
Criminal activity	
Have been arrested	16 (89%)
Have been in prison	7 (39%)
Services	
Currently accessing substance misuse treatment services (community provision; psychosocial, detox and substitute prescribing)	13 (72%)
ATS use^40^	
Current dependent user	7 (39%)
Former dependent user	6 (33%)
Former frequent non-dependent user	2 (11%)
Non-frequent user (current or former)	3 (17%)

### Initiation of ATS use

Participants discussed the initiation of ATS use with reference to a variety of factors, both in terms of strategies to promote or improve positive mental health and wellbeing, as well as functional or preventative strategies to cope with or prevent further deterioration of existing mental health issues. For many individuals, a combination of these were evident, and experiencing co-occurring stressors and chaotic life circumstances further compounded their ability to cope.

A perceived mental health promoting benefit of ATS was improved alertness and increased energy levels, which participants believed allowed them to function better, and to negotiate the demands of their day-to-day lives. Many participants experienced multiple stressors in their lives, and this was one of the main contributing factors in their initiation of ATS use. They normalised their use as a way of as managing childcare (particularly mothers), and employment.

‘I was managing work, full-time employment, with football, with two children’. *(36-year-old male, former dependent user)*

‘I was getting up during the night to feed (the baby) and stuff like that. Then his dad came in one day and he had something, and I just took it’. *(30-year-old female, former dependent user)*

Some participants also spoke about experiencing low self-esteem and a lack of confidence, therefore turning to ATS use to boost their self-belief, and navigation of social situations. They believed that using ATS improved self-concept, and fortified personality traits which they had previously perceived as fragile, making them feel more extraverted, presenting fewer neurotic symptoms and therefore better positioned to cope in their day-to-day lives.

‘The drugs did for me what I couldn’t do for myself. They made me confident. They made me talkative, especially drugs like Ecstasy’. *(38-year-old male, former dependent user)*

‘It was quite good for confidence at first, it’s not until later on you realise what it does’. *(40-year-old male, current dependent user)*

Participants also reported that ATS provided them with an opportunity to escape problems they faced in their lives, which were causing significant distress, when they felt that it was not possible to overcome these issues or find solutions to improve their situation. These concerns included strained relationships with partners and family members, financial worries, as well as issues associated with existing mental ill health. Many participants spoke about being offered ATS from concerned others, such as friends and family members, who suggested it as an alternative way of coping with the chaotic facets of their lives.

‘I heard people take drugs just to escape from the arguing. That’s what I wanted at first, so I started taking it like that’. *(40-year-old male, current dependent user)*

‘Folk take it to hide their feelings, to cover up stuff, just to make themselves feel better because when you’re on drugs, you’re on top of the world. Nothing can hurt you; you feel invincible’. *(21-year-old female, current dependent user)*

Participants referred to the impact of particularly traumatic events in their lives, and how ATS use was something they engaged with to cope with the emotional distress they felt. Many of these traumatic events had occurred recently, such as the dissolution of a romantic or intimate relationship, or bereavement from the loss of a partner or family member. However, participants also referred to the lasting impact of adverse events which had occurred in the past (including adverse childhood experiences), such as traumatic childhood events and historic sexual abuse, and how ATS use lessened the impact of these painful memories on their present-day lives.

‘When our relationship completely broke down, he hung himself. This was the point where my life spiralled out of control’. *(44-year-old female, former dependent user)*

‘I came from obviously a dysfunctional childhood, a dysfunctional family. I was in and out of the care system until I was quite old actually. That would also give me a release from everything I used to think about’. *(40-year-old male, current dependent user)*

### Continued and increased ATS use

When participants discussed factors that encouraged them to continue and potentially increase their use of ATS, they referred to the continued management of multiple stressors in their lives, the perception of apparent positive effects on their mental health and wellbeing, and the need to self-manage deleterious effects of ATS use.

Many participants highlighted the perceived positive effects on their mental health, as a result of using ATS. These included feeling more confident and less anxious, a perceived sense of better life management, and feeling they were better equipped to cope with their day-to-day circumstances. These individuals continued using ATS as an attempt to sustain these desirable effects, which were associated with an overall sense of bolstered wellbeing.

‘I felt like I was invincible, honest to God, I could run five Great North Runs. It took the weight off my shoulders. It like my problems had just gone. I got my confidence. I liked it’. *(41-year-old female, current dependent user)*

‘They made me confident. They made me talkative, especially drugs like Ecstasy and stuff like that’. *(38-year-old male, former dependent user)*

Several participants also described the ongoing management of their ‘chaotic’ lives, the negotiation of multiple individual, social and environmental stressors and deteriorating mental health as motivation for continued ATS use. As their circumstances became increasingly demanding and pressurised, ATS use became increasingly normalised and a part of their daily routine. Over time, most dependent users could not see a way of pursuing their lives without using ATS to support them, even if their ATS was contributing to their deteriorating mental health.

‘I was dealing with the death of my children’s dad, and I think because without it I had no energy at all. Looking back now I was probably very severely depressed, so I was kind of using it as a coping mechanism’. *(44-year-old female, former dependent user)*

‘My mental state just got a bit erratic. I started getting very angry once I’d been up for a couple of days, so my head was just a bit all over the place’. (23*-year-old male, former frequent non-dependent user*)

A dominant theme was self-management of the ‘comedown’ from ATS intoxication. Participants spoke about experiencing worsening psychological symptoms, including low-mood, anxiety, feelings of paranoia, engaging in self-harm behaviours and experiencing thoughts of suicide. This deterioration in mental health led to continued and/or increased ATS use to ‘self-medicate’ these symptoms, even though these issues were often, but not exclusively, their reason for initiating ATS use to begin with. Some participants reported that as their use continued, these symptoms worsened even further, leading them to increase their consumption use both in terms of frequency and quantity, establishing a cycle of maladaptive ATS use behaviour.

‘On a come down and you’re like, fuck my life. Genuinely, that’s what it’s like and then you feel sorry for yourself again for like two days, three days and then you’re fine. Then before you know it, again, another one’s been ordered’. *(21-year-old female, current dependent user)*

‘I’ve used various things in between that, but I always went back to amphetamines. It’s the only thing that’s kept us normal. I’m paranoid when I’m not in it, but when I have it, it takes away the paranoia’. *(40-year-old male, current dependent user)*

### Decreased and remitted ATS use

Participants discussed the negative impacts of continued ATS use, such as a decline in overall mental health functioning, and negative changes to their personality as motivating factors in reducing or ceasing their ATS use. Participants also referred to a desire for change and wanting a ‘better life’. ATS users who had managed to reduce or cease their use spoke about the restoration of ‘normality’ in their lives.

Many participants had previously referred to perceived positive impacts on their wellbeing due to ATS use, including increased confidence. However, after prolonged use several participants described becoming increasingly aware of negative traits and changes to their personas, including paranoia, which they attributed to their ATS use. These perceived changes in personality often motivated a desire to reduce or cease ATS use.

‘Your personality can flip side, you know, popular, sociable, outgoing person and then you’re going to lose the grip on who you are’. (40-year-old male, *former non-frequent user*)

‘I changed in the sense of the drugs I as taking. I wasn’t the nice fella. I became very controlling, very paranoid, really insecure’. (38-year-old male, *former dependent user*)

Despite many participants describing that they initially experienced an alleviation of their negative mental health symptoms from using ATS, after prolonged use these perceived benefits very often became less pronounced, or harder for the user to identify. For those users who reported increasing or uncontrolled use, participants became more focussed on their substance use as a way of alleviating symptoms of mental ill health. Many participants described deleterious impacts on their mental health functioning, as their use increased in terms of both frequency and quantity. This was often cited as a motivating factor in reducing or stopping the use of ATS. However, it was often not until participants reached a point of crisis which resulted in the intervention of support services, before reduced ATS use occurred.

‘I was dead August last year, everything, and no feelings, nothing, soul gone, the lot. You know, it was just black dog’. (40-year-old male, *former non-frequent user*)

‘I was suicidal. I wanted to die. I didn’t want to live, and I hated myself, all that self-loathing and stuff and self-hatred. I thought everybody hated me’. (38-year-old male, *former dependent user*)

Many participants spoke about a breaking point or hitting ‘rock bottom’ as a primary motivating factor in the decision to modify their ATS use. By this stage, participants were unable to associate their ATS use with any benefits or positive effects, and instead apportioned the blame for their negative mental health state and the poor circumstances of their lives to their ATS use. This motivated a desire for change and wanting a ‘better life’.

‘It got to a point where I thought, “I’ve got to sort my head out,” because I’d gotten myself into a bit of a rut’. (40-year-old male, *former dependent user*)

‘For me it was 25, which I’m really grateful for that I hit that rock bottom and I realised I’d had enough because I did’. (38-year-old male, *former dependent user*)

However, for many participants change was an incremental process, and relapse was a common occurrence for participants, with the overall reduction and desistence from use very much associated with building personal resilience over time. Individual, social and environmental triggers often provided the catalyst for relapse and re-engagement with ATS use, followed by a period of reflection and consolidation of goals by the individual user. Participants who had successfully been able to reduce their ATS use, or stop all together, spoke about the restoration of a positive state of mental health and emotional wellbeing; being able to engage in positive everyday activities, which had not been possible amid their most intensive phase of ATS use. Participants were able to re-establish ‘normal’ practices, such as maintaining a regular sleeping pattern, getting up out of bed and ready and leaving the house, which individuals has previously struggled to sustain. This restoration of normality was perceived as a hugely important step forward in participants’ recovery.

‘I can smile. I can brush my hair, put a bit of make-up on. I walked up here. I am going to bed at nine, ten o’clock on a night and I am sleeping all night. Usually, I wouldn’t be able to sleep until three, four o’clock in the morning’. (29-year-old female, *former frequent non-dependent user*)

‘Basically, I’ve resurrected my own mind, body and soul and it took me three and a half months’. (40-year-old male, *former non-frequent user*)

## Discussion

We found that the initiation of ATS use was often initially viewed by participants as a positive strategy for bolstering their wellbeing and confidence, or as a coping mechanism for managing poor mental health and escaping traumatic or challenging issues. Continuation and increase of ATS use were associated with the continued management of multiple stressors in their lives; positive perception of effects on their wellbeing; management of negative side effects and self-medicating to maintain the perceived mental health benefits. Reducing and ceasing ATS use were associated with a decline in overall mental health functioning; negative personality changes; and a desire for change and wanting a ‘better life’. Participants referred to the lasting impact of events which had occurred in the past, and existing research has suggested that experiences of trauma may be an important risk factor for distinguishing individuals who develop substance use issues, from those who do not.^48,49^ This follows existing research that states that age and stage of life may play an important part in determining whether or not individuals are able to use adaptive coping strategies, with users who initiate substance use at an earlier age displaying higher disengagement, lesser use of social support, higher problem avoidance and increased social withdrawal.^50,51^ Participants discussed a range of short-term and prolonged effects on their mental health, including depression and anxiety disorders, which have previously been documented amongst ATS users, related both to the acute effects of intoxication and prolonged effects related to withdrawal.^1,13^

Continued use was perpetuated by the perceived positive influences using ATS was having on individuals’ lives. However, increased ATS use was also associated with the requirement for greater amounts of ATS to elicit similar effects, or to counter negative side effects and ‘come-downs’. Self-medication was initially perceived as beneficial, and there is existing evidence that some users engage in ATS use as a form of self-medication for existing mental ill-health, or to manage symptoms associated with ADHD.^52^ However, many individuals reported their mental health issues worsening due to prolonged ATS use, and often found these effects to be outweighing the positives over time. Participants also referred to adverse changes in their personality with prolonged ATS use, and factors associated with personality type, and the presence of certain personality disorders could provide explanation for increased risk of poor mental health outcomes, and substance use dependency.^53,54^

A desire for a ‘better life’ or ‘normality’ was one of the strongest motivating factors for participants to stop using ATS, and much existing research has focussed on early adulthood as the period when regular and heavy use of substances declines; due to abundant social role transitions during this period, a process known as ‘maturing out’.^55^ However, for some users, it is only when a point of crisis is reached, which serves as a turning point and that there is an opportunity for individuals to break the cycle of substance use and seek support or treatment.^56,57^ The social identity model of recovery proposes that these turning points can constitute the beginning of a process that allows individuals to construct an identity that supports their transition to recovery.^58^ However, by this definition there is a risk for some users that a reduction in ATS use may not occur without a ‘crisis’, which could have other far-reaching impacts on their lives.

In society, ATS are often perceived as safe and recreational substances,^9^ which is a major barrier to the prevention and treatment of problematic use, and to effective public health messaging, which in recent decades has focussed on harm reduction,^59^ rather than scare tactics and fear-based messages, which whilst often drawing criticism, may be effective prevention strategies.^60^ This, in association with medical models which characterise addition as a primary chronic disease of neural circuitry,^35^ focussing on medical solutions to social problems,^61,62^ and a lack of targetted treatment for problematic ATS use, continues to leave affected individuals at disadvantage.^32^ Another concept which may influence help-seeking, and the remittance of ATS use is ‘flourishing’, which proposes that high levels of both hedonic well-being (life-satisfaction, happiness) and eudaimonic well-being (social contribution, purpose in life, personal growth) reduces the risk of mental health and substance use disorders, but only when taking into consideration the impact of life-events and social support.^63^ This is particularly relevant here, as existing research has shown that dependent ATS users experience a greater number of negative life events, and that individuals’ social environment is affected by these negative life events.^64^

This work helps to develop understanding around the complex and bi-directional relationship between ATS use and mental health,^19-21^ and importantly highlights that individuals change their use of substances throughout their drug use careers as both antecedents to and consequences of their mental health. The findings from this study highlight that there is no ‘one size fits all’ approach to prevention and treatment, and rather than focussing on whether it is the mental health or substance use issue which occurred first, it is important to focus on users’ individual circumstances and work to address these issues in co-occurrence.^24,65^

### Strengths and limitations

The principle strength of this work is the qualitative nature of enquiry, which responds to a growing momentum and commitment to include the experiences and views of those whose voices are often overlooked or under-represented.^66^ These findings help to challenge stereotypes about substance use, develop a deeper understanding of hidden populations and behaviours, and further demonstrate that substance use is shaped by a complex set of individual, social, environmental and cultural factors.^29,36^ Whilst the sub-sample was relatively small, it was diverse in terms of age, gender and current or former ATS use status, and recruitment was undertaken via multiple sources, which allowed individuals from a variety of backgrounds to engage with the research and provide rich accounts of their experiences. The ATTUNE study only collected data in the North East of England, an area which has its own cultural dynamics, and economic challenges^37,38,67^; with a higher rate of drug-related deaths (96.3 per million people) than any other region in England or Wales^68,69^; the highest suicide rate in England,^70^ and low levels of ethnic diversity,^71^ which may not make the findings generalisable to other regional contexts, or countries. Whilst we attempted recruitment via organisations representing lesbian, gay, bisexual, transgender, queer/questioning and intersex (LGBTQI) communities, we had no success; and additionally, sexuality and gender identity information about participants was not collected. This is a further limitation of the study, as we know that gay and bisexual men use illicit drugs, including ATS, at higher rates than most other population groups.^72,73^

## Conclusions

Many ATS users lead chaotic lives and engage in multiple risk behaviours, however there is a need to better understand and conceptualise the dynamic interaction between different individual, social, environment and cultural factors that determine individuals’ mental health and substance use trajectories, as they are not a homogenous group.^45-47^ The early identification of issues associated with both mental health and ATS use is universally important, to ensure they do not perpetuate one another, and these findings highlight the need for more joined-up, tailored and holistic approaches to intervention development. A public health approach could be through preventative work in schools, particularly focussing on misconceptions about the safety of ATS; the relationship between mental health and substance use and reducing stigmatisation. Policymakers must also remain mindful that the prevalence of both ATS and mental health issues are higher in more deprived areas, and therefore preventative strategies should be targetted at these areas accordingly. Future research should also engage with varied and diverse population groups; and explore in-depth individuals’ preferences for, and acceptability of treatment opportunities, and the barriers and facilitators to accessing treatment.
